# Blood Transfusion and Survival of Children, Adolescent, and Young Adult Patients with Osteosarcoma: A Multicenter Retrospective Cohort Study

**DOI:** 10.3390/cancers17010097

**Published:** 2024-12-31

**Authors:** Sukjoo Cho, Jamie L. Fierstein, Racha T. Khalaf, John M. Morrison, Jonathan Metts

**Affiliations:** 1 Department of Pediatrics, University of South Florida Morsani College of Medicine, Tampa, FL 33606, USA; sukjoo.cho@emory.edu (S.C.); khalafr@usf.edu (R.T.K.); 2 Aflac Cancer and Blood Disorders Center, Children’s Healthcare of Atlanta and Emory University, Atlanta, GA 30329, USA; 3 Epidemiology and Biostatistics Shared Resource, Johns Hopkins All Children’s Institute for Clinical and Translational Research, Johns Hopkins All Children’s Hospital, St. Petersburg, FL 33701, USA; jamie@jhmi.edu; 4 Department of Anesthesiology and Critical Care Medicine, Johns Hopkins University School of Medicine, Baltimore, MD 21205, USA; 5 Division of Pediatric Hospital Medicine, Johns Hopkins All Children’s Hospital, St. Petersburg, FL 33701, USA; jmorri86@jhmi.edu; 6 Department of Pediatrics, Johns Hopkins University School of Medicine, Baltimore, MD 21287, USA; 7 Cancer and Blood Disorders Institute, Johns Hopkins All Children’s Hospital, St. Petersburg, FL 33701, USA; 8 Sarcoma Department, H. Lee Moffitt Cancer Center and Research Institute, Tampa, FL 33612, USA

**Keywords:** osteosarcoma, transfusion, blood, survival

## Abstract

**Background/Objectives**: Prior studies suggest that blood transfusion may adversely affect the survival of patients with cancer via transfusion-related immunomodulation. The objective of our study is to investigate the association between transfusion during neoadjuvant chemotherapy and survival in children, adolescent, and young adult (CAYA, 39 years old or younger) patients with osteosarcoma. **Methods**: This is a multicenter retrospective cohort study of patients between 2007 and 2022. Our primary exposure was receipt of any blood product in the neoadjuvant period (i.e., neoadjuvant transfusion). The primary outcome of interest was 3-year event-free survival (EFS) calculated using the Kaplan–Meier method, while secondary outcomes of interest included 5-year EFS and 3- and 5-year overall survival (OS). Firth multivariable logistic regression models were constructed to evaluate the adjusted association between transfusion status and 3- and 5-year EFS and OS. **Results**: In total, 73 patients were included in the analytic sample; among them, 34 received neoadjuvant transfusion. There was no significant difference between transfused and non-transfused groups in race, ethnicity, tumor location, stage at diagnosis, histologic response to neoadjuvant chemotherapy, and receipt of ifosfamide or radiation during initial treatment. The transfusion group included more females (*p* = 0.02) and lower median hemoglobin at diagnosis (*p* = 0.002) than the non-transfusion group. EFS and OS did not significantly vary by transfusion status or type. **Conclusions**: We did not observe an adjusted association between neoadjuvant transfusion and survival in CAYA patients with osteosarcoma.

## Simple Summary

Although blood transfusion is generally considered lifesaving, there have been long-standing concerns that it may suppress the anti-cancer immunity of recipients. The aim of our study is to assess the potential effect of transfusion on the survival of children, adolescent, and young adult (CAYA, 39 years old or younger) patients with osteosarcoma, the most common malignant bone tumor. We focused on blood transfusion during chemotherapy before surgery, when the tumor remains bulky with continued risk for metastasis. Our data did not reveal any association between presurgical transfusion and the survival of CAYA patients with osteosarcoma. We detected no specific difference by transfusion type (red blood cells, platelets). Our findings provide reassurance given decades-long discussions about transfusion-related suppression of anti-cancer immunity.

## 1. Introduction

Osteosarcoma is the most common malignant bone tumor with an estimated 1000 new diagnoses per year in the United States [1,2]. It primarily occurs in children, adolescent, and young adult (CAYA, 39 years old or younger) patients with a peak incidence in adolescence. The most common tumor location is long bones including the femur, tibia, and humerus, although the disease can happen in any bone of the body. Approximately 20% of new cases are metastatic at presentation, most frequently to the lung [3]. The standard treatment for osteosarcoma consists of tumor resection with neoadjuvant and adjuvant chemotherapy with high-dose methotrexate, doxorubicin, and cisplatin, which has remained largely unchanged for more than 30 years [4,5]. Although the 5-year event-free survival (EFS) of patients with localized disease exceeds 60%, the 5-year EFS of those with metastatic disease remains poor at <20% [1,4]. Tumor size, location, metastasis at diagnosis, histologic response to neoadjuvant chemotherapy, and surgical remission are independent prognostic factors in osteosarcoma [6,7].

While transfusion is common in patients with osteosarcoma due to the marrow-suppressive effect of chemotherapy, the association of blood transfusion with disease prognosis remains unclear. The relationship between transfusion and survival in patients with cancer has been a long-standing discussion since the initial discovery of transfusion-related immunomodulation (TRIM) in the 1970s [8,9]. While the exact mechanism is obscure, it has been speculated that transfusion can dampen the recipient’s immune system and subsequently promote the growth of malignant cells. This topic has been extensively investigated in certain malignancies such as colorectal cancer and lung cancer [10,11,12,13,14,15,16,17,18,19,20,21,22]. However, there are limited existing data on the association of transfusion with the survival of patients with osteosarcoma. We thus aimed to evaluate the association between transfusion during neoadjuvant chemotherapy and the EFS and overall survival (OS) of CAYA patients with osteosarcoma. Since most patients with osteosarcoma receive transfusion at some point during their treatment, we chose to focus on the neoadjuvant phase, when the tumor burden is highest during initial therapy and metastatic potential remains before local control. Our speculation was that this potential could be amplified by dysfunctional anti-tumor immunity secondary to TRIM.

## 2. Materials and Methods

### 2.1. Study Design and Population

We conducted a multicenter retrospective analysis of CAYA patients with osteosarcoma treated at Johns Hopkins All Children’s Hospital in St. Petersburg, FL, from January 2007 to December 2022 and Johns Hopkins Hospital in Baltimore, MD, from November 2016 to December 2022. The discrepant recruitment dates between the two institutions were because of the start dates of the current electronic health record (EHR) platforms in each center and the availability of legacy EHR data at Johns Hopkins All Children’s Hospital. The study was approved by the institutional review board at Johns Hopkins All Children’s Hospital (IRB00318885). Our analysis was performed in accordance with the Strengthening the Reporting of Observational Studies in Epidemiology (STROBE) guidelines (Table S1) [23].

Due to the lack of a single diagnostic code for osteosarcoma, search queries were built using eligibility criteria consisting of (1) diagnostic codes for bone cancer (C40 or C41) and (2) a medication code for cisplatin (2555) as per the International Classification of Diseases 10th Revision (ICD10). Cisplatin is a standard-of-care first-line chemotherapy agent for osteosarcoma but used less commonly for other bone cancers. Identified patients were then manually reviewed to confirm the diagnosis of osteosarcoma. Our exclusion criteria included an unclear diagnosis date, comorbidity that required frequent transfusion [e.g., Diamond–Blackfan anemia (DBA)], and/or a considerable portion of treatment occurring at an outside institution, thereby precluding our ability to observe their transfusion history. Two reviewers (S.C. and J.M.) independently performed manual reviews of all identified patients.

### 2.2. Study Exposures

The primary exposure was neoadjuvant transfusion (i.e., administration of any blood product after diagnosis and before surgical resection of the primary site, which typically takes place at 10 weeks of treatment) defined with a binary variable (transfusion/no transfusion). Perioperative transfusion was not included. All blood products underwent leukoreduction and irradiation during preparation as per institutional standards for oncology patients. Additional study exposure was transfusion type (e.g., red blood cells (RBCs), platelets).

### 2.3. Outcomes of Interest

Our primary outcome of interest was 3-year EFS. The secondary outcomes of interest were 5-year EFS and 3- and 5-year OS. EFS was defined as the time from the date of diagnosis (i.e., diagnostic biopsy or surgery of the primary tumor) to the first event (i.e., local recurrence, new metastasis, progression of existing metastasis, second malignancy, death, or a combination of these events). OS was defined as the time from the date of diagnosis to the date of death. If a patient did not die at the date of data cut-off for the analysis, the patient was considered censored at the time of the last follow-up before the cut-off date.

### 2.4. Other Parameters of Interest

Demographic and clinical characteristics including age, sex, race, ethnicity, location of the primary tumor, stage at diagnosis, hemoglobin at diagnosis, histologic response to neoadjuvant chemotherapy, exposure to ifosfamide, exposure to radiation during first-line chemotherapy, and osteosarcoma occurring as a secondary malignancy were collected via chart review. Histologic response to neoadjuvant chemotherapy was categorized as good (>90% necrosis) and poor (≤90%) by convention for osteosarcoma [24]. Ifosfamide exposure was collected because it is a chemotherapy agent with significant myelosuppressive effects that was studied in the past for patients with osteosarcoma with high-risk features (e.g., poor necrosis, metastatic disease) [4,24,25].

### 2.5. Statistical Analyses

Demographic and clinical characteristics as well as the total number of transfusions during osteosarcoma treatment were compared by neoadjuvant transfusion status (yes/no). Continuous variables were reported as medians with minimum to maximum ranges and categorical variables with numbers and percentages. To determine differences between transfusion groups, Mann–Whitney *U* tests and χ^2^ or Fisher’s exact tests were used for continuous variables and categorical variables, respectively. Kaplan–Meier EFS and OS estimates at 3 and 5 years were calculated along with corresponding 95% confidence intervals (CIs). Survival functions were compared with two-sided log-rank tests while pointwise survival rates were evaluated with two-sided z-tests of proportion accounting for right-censored data. Additionally, we constructed multivariable Firth logistic regression models to estimate the adjusted association between neoadjuvant transfusion status and odds for 3- and 5-year EFS (yes/no) or OFS (yes/no). Firth logistic regression is a penalized likelihood-based method that provides a potential reduction in bias for small samples and/or rare outcomes such as those observed in this study [26,27]. Models were adjusted for an a priori-determined group of covariates previously demonstrated to be associated with the outcomes among this study population, including sex and cancer stage [28,29,30,31]. Histologic response to neoadjuvant chemotherapy was initially considered as a covariate; however, the sample sizes were extremely limited and concern for artificially inflated estimates prompted their eventual exclusion from the final models. Adjusted odds ratios (ORs) and 95% CIs were visualized with forest plots. Missing data were not imputed and two-sided *p*-values < 0.05 were considered statistically significant. All statistical analyses were performed using Stata SE Version 17.1 (Stata Corp LP, College Station, TX, USA).

## 3. Results

**Figure 1:**
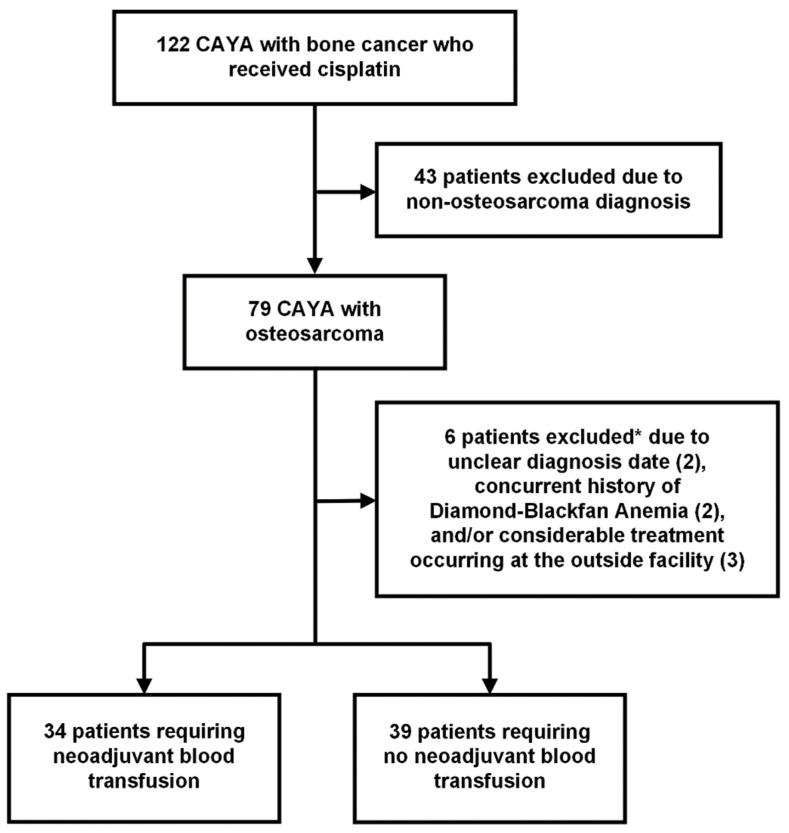
Study flowchart. * One patient excluded due to both unclear diagnosis date and history of Diamond-Blackfan anemia.

The initial search identified 122 CAYA patients diagnosed with bone cancer who received cisplatin as a first-line treatment (Figure 1). Forty-three patients were manually excluded due to non-osteosarcoma diagnosis, and six additional patients were excluded due to unclear diagnosis date, comorbidity of DBA, and/or substantial treatment occurring at an outside facility. The final analytic sample consisted of 73 patients, including 34 patients who received neoadjuvant transfusion and 39 patients who did not. The number of patients who received care at Johns Hopkins All Children’s Hospital was 54, while that at Johns Hopkins Hospital was 19.

Table 1 displays demographic and clinical characteristics of the identified patients by neoadjuvant transfusion status. There was no significant difference in age at diagnosis, race and ethnicity, use of ifosfamide or radiation as first-line treatment, stage at diagnosis, tumor location, histologic response to neoadjuvant chemotherapy, and secondary malignancy between transfused and non-transfused groups. Notably, we found significant differences in sex at birth (*p* = 0.02) and hemoglobin at diagnosis (*p* = 0.002) between groups. The male-to-female ratio was 1:1 among transfused patients and 3:1 in non-transfused patients. The median (minimum to maximum range [range]) hemoglobin at diagnosis of transfused and non-transfused patients was 12.4 (9.7 to 14.7) and 13.9 (8.8 to 16) g/dL, respectively. In addition, we observed a marked gap in hemoglobin at diagnosis according to sex at birth. The median hemoglobin at diagnosis was 12.3 g/dL in females, 1.7 g/dL lower than that of males (*p* = 0.002, shown in Table S2).

Among 34 patients receiving neoadjuvant transfusion, 30 (88.2% of the transfused patients) received RBCs and 14 (41.2%) received platelets, while 10 (29.4%) received both RBCs and platelets. The median (range) instance of neoadjuvant transfusion was 1 (1 to 6). Half of the transfused patients required any blood product once during the neoadjuvant period.

The median (range) follow-up time for the entire cohort was 2.9 (0.5 to 11.4) years. The median (range) follow-up time for the transfused and non-transfused groups was 3.8 (0.8 to 11.4) and 2.8 (0.5 to 6.4) years, respectively.

**Figure 2:**
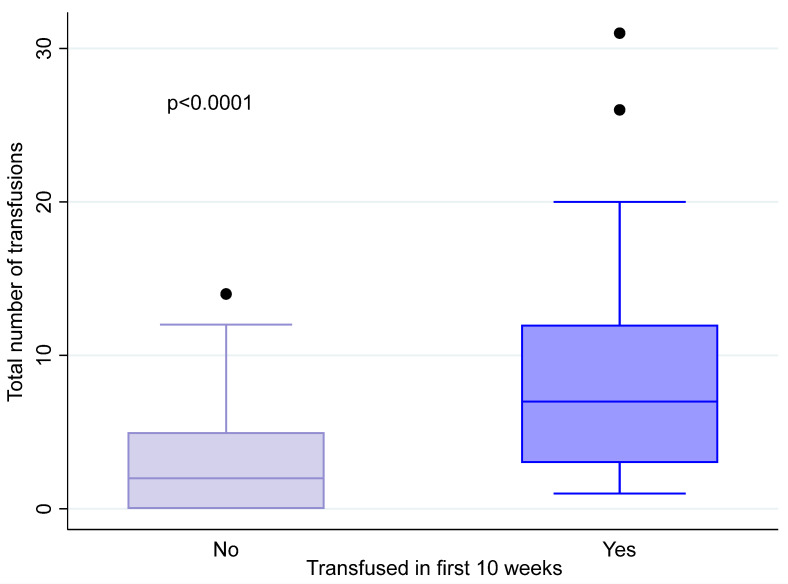
Box plot of total number of transfusions during osteosarcoma treatment by neoadjuvant transfusion status.

Of note, neoadjuvant transfusion was associated with further transfusion needs during osteosarcoma treatment. As shown in Figure 2, we detected a significant difference in the total number of transfusions required throughout treatment by neoadjuvant transfusion status (*p* < 0.0001). The median (range) total transfusion instance of patients who required neoadjuvant transfusion was 7 (1 to 31), whereas that of patients who did not was n = 2 (0 to 14). There were 13 (1.9%) patients who did not require any blood product throughout their treatment course.

**Figure 3:**
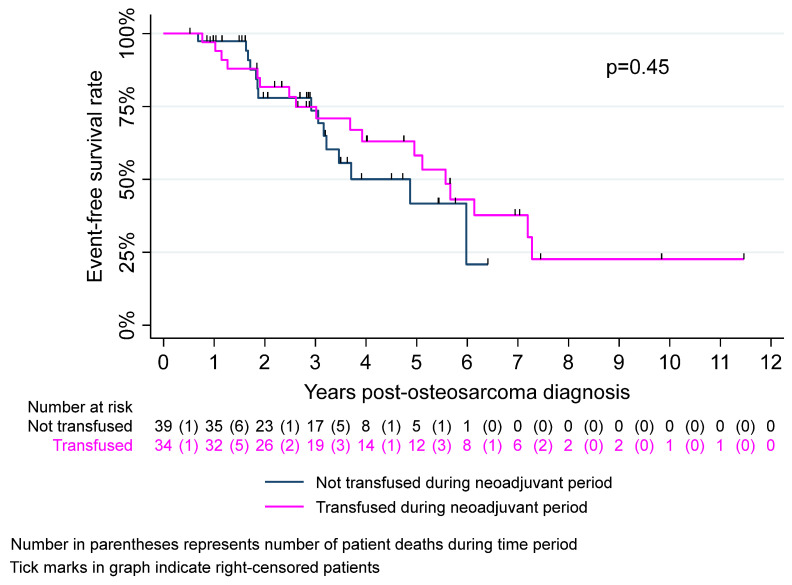
Kaplan–Meier event-free survival estimates by neoadjuvant transfusion status.

**Figure 4:**
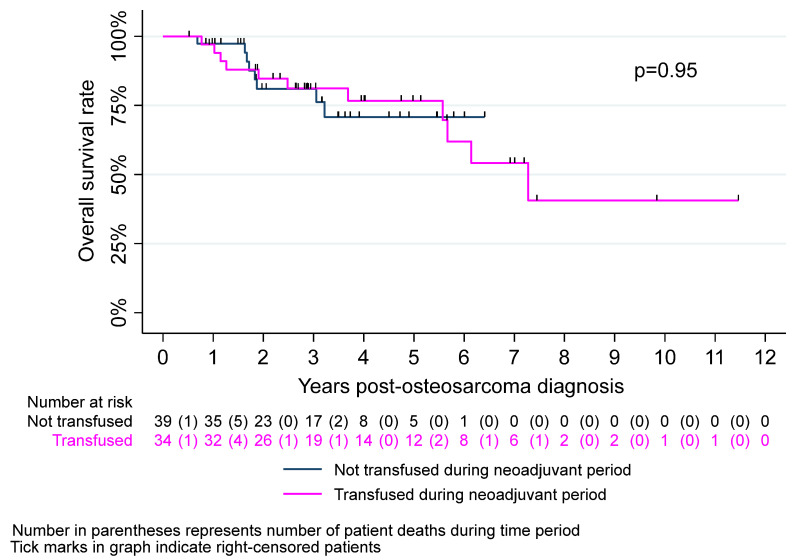
Kaplan–Meier overall survival estimates by neoadjuvant transfusion status.

Table 2 summarizes survival outcomes of CAYA patients with osteosarcoma by neoadjuvant transfusion status. We observed no significant difference in EFS between the transfused and non-transfused groups (*p* = 0.92). Three-year EFS was approximately 74% in both groups. EFS at 5 years was 58% and 42% in the transfused and non-transfused groups, respectively (*p* = 0.60). There were no significant intergroup differences in 3- or 5-year OS (*p* = 0.99 and *p* = 0.77, respectively). Kaplan–Meier curves for EFS and OS are described in Figure 3 and Figure 4, respectively.

**Figure 5:**
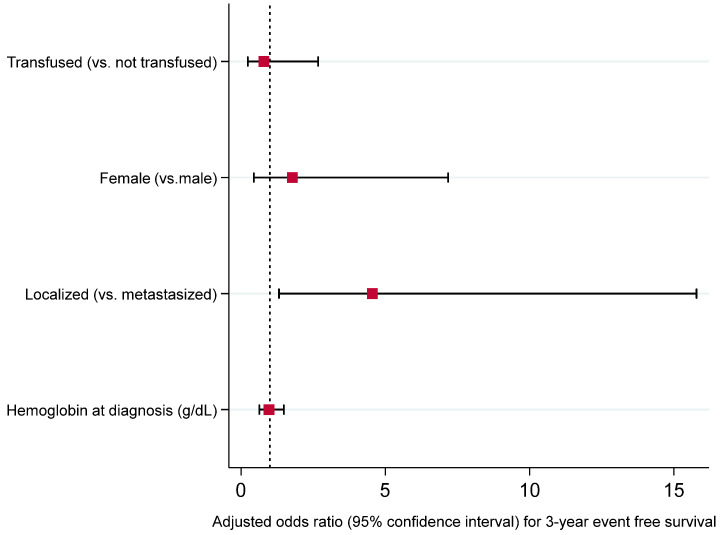
Forest plot visualizing adjusted odds ratios and 95% confidence intervals of variables included into a Firth logistic regression model for 3-year event-free survival (dashed line, odds ratio of 1; bullets, odds ratios; whiskers, 95% confidence intervals). Detailed results presented in Table S3.

**Figure 6:**
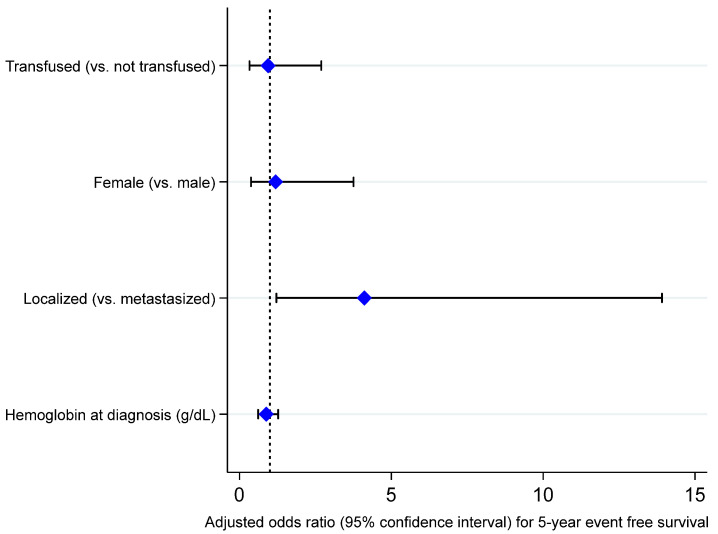
Forest plot visualizing adjusted odds ratios and 95% confidence intervals of variables included in a Firth logistic regression model for 5-year event-free survival (dashed line, odds ratio of 1; bullets, odds ratios; whiskers, 95% confidence intervals). Detailed results presented in Table S4.

**Figure 7:**
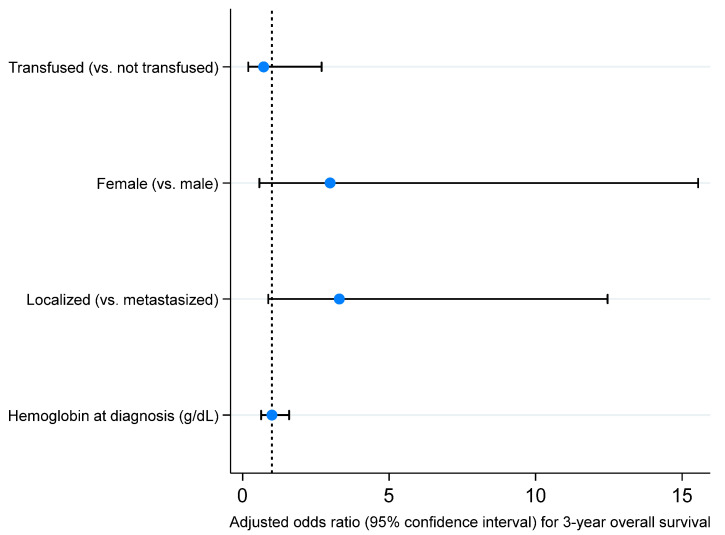
Forest plot visualizing adjusted odds ratios and 95% confidence intervals of variables included in a Firth logistic regression model for 3-year overall survival (dashed line, odds ratio of 1; bullets, odds ratios; whiskers, 95% confidence intervals). Detailed results presented in Table S5.

**Figure 8:**
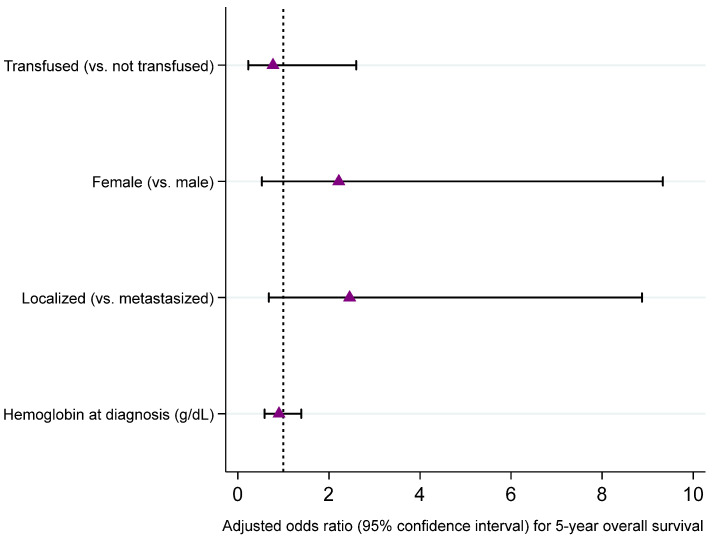
Forest plot visualizing adjusted odds ratios and 95% confidence intervals of variables included in a Firth logistic regression model for 5-year overall survival (dashed line, odds ratio of 1; bullets, odds ratios; whiskers, 95% confidence intervals). Detailed results presented in Table S6.

To evaluate the adjusted association between neoadjuvant transfusion and survival outcomes, we performed Firth multivariable logistic regression including sex at birth, stage at diagnosis, and hemoglobin level at diagnosis as covariates. As seen in Figure 5, Figure 6, Figure 7 and Figure 8, we did not observe an adjusted association between neoadjuvant transfusion status and any of the survival outcomes in the final multivariable models. The analyses demonstrated that patients with localized disease had greater odds for 3- and 5-year event-free survival compared to patients with metastasis (3-year EFS OR: 4.6, 95% CI: 1.3 to 15.8; 5-year EFS OR: 4.1, 95% CI: 1.2 to 13.9).

As shown in Table 3, we conducted subgroup survival analysis by transfusion type. Among the entire cohort, 41% required RBC transfusion during the neoadjuvant period. Three-year EFS of this group was 76% and did not differ significantly from 3-year EFS of those not requiring RBC (*p* = 0.86). Fewer patients required neoadjuvant platelet transfusion (n = 14, 19.2%) than RBC transfusion (n = 30, 41.1%). Our analysis did not reveal significant differences in survival outcomes by subgroup.

## 4. Discussion

In this multicenter retrospective study, neoadjuvant transfusion was not associated with the EFS and OS of CAYA patients with osteosarcoma. We did not observe any specific difference by transfusion type. Our findings were reassuring given decades-long concerns for TRIM and associated cancer progression and recurrence.

Existing data to provide context to our findings are limited. The only prior study to date that investigated the relationship between blood transfusion and survival in patients with osteosarcoma was reported by Chesi and colleagues in 1989 [32]. This retrospective analysis included 155 patients with localized osteosarcoma of the long bones who were treated with amputation followed by adjuvant chemotherapy. The study demonstrated evident associations of perioperative blood transfusion with increased metastasis and decreased survival time. Specifically, 3-year disease-free survival (i.e., survival without metastasis) was approximately 36% and 59% in transfused and non-transfused patients, respectively (*p* < 0.05). OS also differed significantly in the two groups. Moreover, the study described that the number of units transfused was significantly associated with disease-free and overall survival.

Unlike our study, Chesi and colleagues’ analysis included patients within the age range of 5 to 59 years that might have had comorbidities affecting both transfusion needs and survival outcomes. In addition, the analysis focused on transfusion in the perioperative timeframe, which is significantly shorter than the neoadjuvant period (i.e., initial 10 weeks of treatment) and might be prone to confounding effects of perioperative complications. Notably, upfront tumor resection with adjuvant chemotherapy was the standard treatment for osteosarcoma when the study was conducted.

Our study focused on neoadjuvant transfusion because most patients with osteosarcoma receive transfusion at some point during their treatment course. Given that the tumor burden is at its peak before local control and represents persistent metastatic risk, we speculated that neoadjuvant transfusion would be critical when evaluating TRIM. Interestingly, neoadjuvant transfusion was a crucial predictor of total transfusion needs during treatment. This finding implies that cumulative effects of transfusions throughout treatment can be reasonably estimated by analyzing data from the neoadjuvant phase. To our knowledge, there are no published data that specifically investigate transfusion during the neoadjuvant period.

Our findings could be explained, at least in part, by evolving techniques of blood product modifications. Since Chesi and colleagues published their data, leukoreduction and irradiation have become routine processes when blood components for patients with cancer are prepared [33,34,35,36,37]. They can eliminate potential mediators of TRIM, which include residual leukocytes in the blood products and their cytokines such as transforming growth factor-beta (TGF-β) [38]. Particularly, robust preclinical evidence has shown that TGF-β promotes progression and metastasis and suppresses immune responses in the tumor microenvironment of various cancers including osteosarcoma [39,40,41].

However, our understanding of TRIM’s exact mechanism remains incomplete. Studies have proposed mediators that can remain after leukoreduction and irradiation, including RBC storage lesions (i.e., changes in erythrocytes in stored RBCs resulting in hemolysis during storage and after transfusion), extracellular vesicles, and iron [42,43,44,45,46,47]. In addition, there is preclinical evidence that platelets and their byproducts (e.g., platelet-derived growth factors) may promote intratumoral angiogenesis, protect circulating tumor cells, and facilitate extravasation and intravasation of tumor cells [48]. At the clinical level, many reports described the association between thrombocytosis and shorter disease-specific survival in various cancers [49,50,51,52,53].

Notably, we observed that female patients required significantly more transfusions compared to their male counterparts. This finding aligns with prior data that female patients developed worse chemotherapy-induced hematologic toxicity during osteosarcoma treatment, requiring more transfusion support and hospitalization for neutropenic fever [54]. Our finding that females had lower hemoglobin at diagnosis than males, presumably due to menstrual cycles, may partly explain increased transfusion needs among females. Despite the observed difference, however, the median hemoglobin at diagnosis of both males and females was above 12 g/dL, notably higher than the typical RBC transfusion threshold (i.e., hemoglobin of 7 g/dL or lower). Furthermore, we found that females did not have decreased survival, suggesting against the negative effect of TRIM on osteosarcoma survival. This is consistent with prior large database studies reporting that female patients with osteosarcoma had better survival than their male counterparts, although the rationale behind this finding remains unclear [28,29].

Our study has several limitations. First, although we attempted to mitigate the small sample size by collecting data from multiple centers, osteosarcoma is a rare disease, and the study power is notably limited. Therefore, a more subtle effect may be present, but undetected, in the study population. Second, given power limitations, we could not perform a transfusion number dependence analysis, which might provide insight into the associations in question. Third, the small patient sample treated at the centers in this study may not be generalizable to the entire osteosarcoma population. Fourth, although we found significant associations in multivariable models, the estimates were relatively imprecise (i.e., wide confidence intervals) given the small sample size. Finally, the retrospective nature of the study relied on EHR data; therefore, it is possible that specific patient data of relevance to our analysis were underreported and/or incorrectly documented.

## 5. Conclusions

In this multicenter retrospective cohort study investigating the association between post-diagnosis neoadjuvant blood transfusion and survival in CAYA patients with osteosarcoma, we observed no association of all-type blood transfusion with 3- and 5-year EFS and OS. RBCs were more frequently needed in females whose hemoglobin at diagnosis was lower than that of males, possibly due to menstrual cycles. However, such increased transfusion needs were not associated with shorter survival. Our findings are reassuring and should provide clinicians with confidence that appropriate transfusion support will not adversely affect patient outcomes. Given the limitations of the small-sample, retrospective analysis, future studies using a large-scale registry or a prospective cohort are warranted. Preclinical efforts to understand the mechanism of TRIM and identify biomarkers could be groundbreaking.

## Figures and Tables

**Table 1:** Demographic and clinical characteristics by neoadjuvant transfusion status.

Characteristic, Statistic	Total (n = 73)	Transfusion (n = 34)	No Transfusion (n = 39)	*p*-Value
**Age at diagnosis, median (range)**	15 (4 to 29)	14 (4 to 29)	15 (9 to 28)	0.50
**Sex, n (%)**				
Male	45 (61.6)	16 (47.1)	29 (74.4)	0.02
Female	28 (38.4)	18 (52.9)	10 (25.6)	
**Race, n (%)**				
Black or African American	12 (16.4)	5 (14.7)	7 (18.0)	0.40
White	50 (68.5)	27 (79.4)	23 (59.0)	
Asian	3 (4.1)	1 (2.9)	2 (5.1)	
Native Hawaiian or Other Pacific Islander	1 (1.4)	0 (0)	1 (2.6)	
Multiracial	2 (2.7)	0 (0)	2 (5.1)	
Unknown/Missing	3 (4.1)	1 (2.9)	2 (5.1)	
**Ethnicity, n (%)**				
Hispanic/Latinx	12 (16.4)	4 (11.8)	8 (20.5)	0.52
Non-Hispanic/Latinx	39 (53.4)	18 (52.9)	21 (53.9)	
Unknown/Missing	22 (30.1)	12 (35.3)	10 (25.6)	
**Mortality during study period, n (%)**	19 (26.0)	11 (32.4)	8 (20.5)	0.25
Hemoglobin at diagnosis, median (range) *	13.3 (8.8 to 16)	12.4 (9.7 to 14.7)	13.9 (8.8 to 16)	0.002
**Ifosfamide as first-line chemotherapy, n (%)**				
Yes	4 (5.5)	3 (8.8)	1 (2.6)	0.33
No	69 (94.5)	31 (91.2)	38 (97.4)	
**Radiation during initial treatment, n (%)**				
Yes	3 (4.1)	2 (5.9)	1 (2.6)	0.59
No	70 (95.9)	32 (94.1)	38 (97.4)	
**Stage at osteosarcoma diagnosis, n (%)**				
Localized	59 (80.8)	26 (76.5)	33 (84.6)	0.38
Metastatic	14 (19.2)	8 (23.5)	6 (15.4)	
**Location of osteosarcoma, n (%)**				
Extremity	68 (93.2)	31 (91.2)	37 (94.9)	0.66
Non-extremity	5 (6.9)	3 (8.8)	2 (5.1)	
**Histologic response to neoadjuvant chemotherapy, n (%)**				
Good	36 (49.3)	16 (47.1)	20 (51.3)	0.94
Poor	31 (42.5)	15 (44.1)	16 (41.0)	
Unknown/Missing	6 (8.2)	3 (8.8)	3 (7.7)	
**Secondary malignancy, n (%)**				
Yes	2 (2.7)	0 (0)	2 (5.1)	0.50
No	71 (97.3)	34 (100)	37 (94.9)	
**Transfusion type, n (%)**				
Platelets only	-	4 (11.8)	-	
Red blood cells only	-	20 (58.2)	-	
Platelets and red blood cells	-	10 (29.4)	-	
**Transfusion instance, n (%)**				
1	-	17 (50)	-	
2	-	7 (20.6)	-	
3	-	3 (8.8)	-	
4+	-	7 (20.6)	-	
**Transfusion instance, median (range)**	-	1 (1 to 6)	-	

Percentages may not add to 100% due to rounding. * One patient excluded due to lack of hemoglobin at diagnosis reported.

**Table 2:** Three- and five-year event-free and overall survival rates by neoadjuvant transfusion status.

Survival Outcome	Survival Rate (95% CI)			*p*-Value
	Total (n = 73)	Transfusion (n = 34)	No Transfusion (n = 39)	
**Event-free survival**				
3-year	74.1% (61.0% to 83.3%)	74.9% (55.9% to 86.6%)	73.6% (53.6% to 86%)	0.92
5-year	52.2% (36.9% to 65.5%)	58.2% (37.5% to 74.2%)	41.7% (19.7% to 62.5%)	0.60
**Overall survival**				
3-year	81.0% (68.9% to 88.8%)	81.2% (62.7% to 91.1%)	81.0% (62.4% to 91.0%)	0.99
5-year	75.7% (61.7% to 85.1%)	76.7% (56.6% to 88.3%)	70.8% (48.7% to 84.7%)	0.77

CI, confidence interval.

**Table 3:** Three- and five-year event-free and overall survival rates by neoadjuvant transfusion status and type.

Survival Outcome	Survival Rate (95% CI)		*p*-Value	Survival Rate (95% CI)		*p*-Value
	RBC Transfusion (n = 30)	No RBC Transfusion (n = 43)		Platelet Transfusion (n = 14)	No Platelet Transfusion (n = 59)	
**Event-free survival**						
3-year	75.6% (55.5% to 87.6%)	73.1% (54.3% to 85.1%)	0.86	91.7% (53.9% to 98.8%)	70.1% (55.0% to 81.0%)	0.17
5-year	62.3% (40.7% to 77.9%)	37.5% (16.6% to 58.4%)	0.35	61.1% (25.5% to 83.8%)	49.1% (32.4% to 63.9%)	0.65
** Overall survival **						
3-year	79.4% (59.7% to 90.2%)	82.6% (65.2% to 91.8%)	0.81	100%	76.8% (62.6% to 86.2%)	0.09
5-year	74.5% (53.2% to 87.1%)	73.4% (52.6% to 86.1%)	0.96	90% (47.3% to 98.5%)	70.4% (54.3% to 81.7%)	0.39

CI, confidence interval; RBC, red blood cell.

## Data Availability

The original contributions presented in this study are included in the article/Supplementary Materials. Further inquiries can be directed to the corresponding author.

## Supplementary Materials

The following supporting information can be downloaded at https://www.mdpi.com/article/10.3390/cancers17010097/s1, Table S1: Checklist per STROBE guidelines for observational studies; Table S2: Hemoglobin at diagnosis by sex at birth; Table S3: Odds ratios and 95% confidence intervals of variables entered into a Firth logistic regression model for 3-year event-free survival; Table S4: Odds ratios and 95% confidence intervals of variables entered into a Firth logistic regression model for 5-year event-free survival; Table S5: Odds ratios and 95% confidence intervals of variables entered into a Firth logistic regression model for 3-year overall survival; Table S6: Odds ratios and 95% confidence intervals of variables entered into a Firth logistic regression model for 5-year overall survival.
