# Supplementary material for: Blood Transfusion and Survival of Children, Adolescent, and Young Adult Patients with Osteosarcoma: A Multicenter Retrospective Cohort Study
Source: Cancers (Basel). 2024 Dec 31;17(1):97. doi: 10.3390/cancers17010097 (PMC11719514; doi:10.3390/cancers17010097)
Supplement: Supplementary file 1 [file cancers-17-00097-s001.zip › cancers-3377458-supplementary.pdf]

Supplementary Materials

Table S1. Checklist per STROBE guidelines for observational studies.

	Item No.	Recommendation	Page No.	Relevant text from manuscript
**Title and abstract**	1	(*a*) Indicate the study's design with a commonly used term in the title or the abstract	1	"A Multicenter Retrospective Cohort Study"
		(*b*) Provide in the abstract an informative and balanced summary of what was done and what was found	1	Abstract was structured as Background/Objectives, Methods, Results, and Conclusions.
**Introduction**				
Background/rationale	2	Explain the scientific background and rationale for the investigation being reported	2	Paragraphs 1-2 of Introduction
Objectives	3	State specific objectives, including any prespecified hypotheses	2	Paragraph 2 of Introduction
**Methods**				
Study design	4	Present key elements of study design early in the paper	2	*Study Design and Population* paragraph of Materials and Methods
Setting	5	Describe the setting, locations, and relevant dates, including periods of recruitment, exposure, follow-up, and data collection	2-3	*Study Design and Population* and *Study Exposures* paragraphs of Materials and Methods
Participants	6	(*a*) Give the eligibility criteria, and the sources and methods of selection of participants. Describe methods of follow-up	2	*Study Design and Population* paragraph of Materials and Methods
		(*b*) For matched studies, give matching criteria and number of exposed and unexposed	N/A	N/A
Variables	7	Clearly define all outcomes, exposures, predictors, potential confounders, and effect modifiers. Give diagnostic criteria, if applicable	3	*Study Exposures* paragraph of Materials and Methods
Data sources/ measurement	8 *	For each variable of interest, give sources of data and details of methods of assessment (measurement). Describe comparability of assessment methods if there is more than one group	3	*Study Exposures* paragraph of Materials and Methods

Bias	9	Describe any efforts to address potential sources of bias	2	"Two reviewers (S.C. and J.M.) independently performed manual reviews of all identified patients."
Study size	10	Explain how the study size was arrived at	3	We collected all available patients qualifying eligibility criteria to maximize the sample size.
Quantitative variables	11	Explain how quantitative variables were handled in the analyses. If applicable, describe which groupings were chosen and why.	3	*Study Exposures* paragraph of Materials and Methods
Statistical methods	12	(*a*) Describe all statistical methods, including those used to control for confounding	3-4	*Statical Analyses* paragraph of Materials and Methods
		(*b*) Describe any methods used to examine subgroups and interactions	3-4	*Study Exposures* paragraph of Materials and Methods
		(*c*) Explain how missing data were addressed	3-4	*Study Exposures* paragraph of Materials and Methods
		(*d*) If applicable, explain how loss to follow-up was addressed	3-4	*Study Exposures* paragraph of Materials and Methods
		(*e*) Describe any sensitivity analyses	N/A	N/A
**Results**				
Participants	13 *	(a) Report numbers of individuals at each stage of study—e.g., numbers potentially eligible, examined for eligibility, confirmed eligible, included in the study, completing follow-up, and analysed	4	Figure 1 and paragraph 1 of Results
		(b) Give reasons for non-participation at each stage	4	Figure 1 and paragraph 1 of Results
		(c) Consider use of a flow diagram	4	Figure 1
Descriptive data	14 *	(a) Give characteristics of study participants (e.g., demographic, clinical, social) and information on exposures and potential confounders)	4-6	Table 1 and paragraphs 2-4 of Results
		(b) Indicate number of participants with missing data for each variable of interest	4	Figure 1 and paragraph 1 of Results
		(c) Summarise follow-up time (e.g., average and total amount)	5	Paragraph 4 of Results
Outcome data	15 *	Report numbers of outcome events or summary measures over time	6-8	Tables 2 and 3, Figures 3 and 4 and paragraphs 6 and 7 of Results

Main results	16	(*a*) Give unadjusted estimates and, if applicable, confounder-adjusted estimates and their precision (e.g., 95% confidence interval). Make clear which confounders were adjusted for and why they were included	6-7, 11	Tables 2 and 3
		(*b*) Report category boundaries when continuous variables were categorized	N/A	N/A
		(*c*) If relevant, consider translating estimates of relative risk into absolute risk for a meaningful time period	N/A	N/A
Other analyses	17	Report other analyses done—e.g., analyses of subgroups and interactions, and sensitivity analyses	6-11	Figures 2 and 5-8, Table 3, and paragraphs 5, 7 and 8 of Results
**Discussion**				
Key results	18	Summarise key results with reference to study objectives	11	Paragraph 1 of Discussion
Limitations	19	Discuss limitations of the study, taking into account sources of potential bias or imprecision. Discuss both direction and magnitude of any potential bias	12	Paragraph 8 of Discussion
Interpretation	20	Give a cautious overall interpretation of results considering objectives, limitations, multiplicity of analyses, results from similar studies, and other relevant evidence	11-12	Paragraph 1-7 of Discussion
Generalisability	21	Discuss the generalisability (external validity) of the study results	12	Conclusions
**Other information**				
Funding	22	Give the source of funding and the role of the funders for the present study and, if applicable, for the original study on which the present article is based	13	"This research was funded by the Johns Hopkins All Children's Foundation (90108593)."

* Give information separately for exposed and unexposed groups.

Table S2. Hemoglobin at diagnosis by sex at birth.

Hemoglobin at diagnosis *	N	Median	25th percentile	75th percentile
Male	45	14	12.8	14.3
Female	27	12.3	11	13.2
Total	72	13.3	12.05	14.2

* p=.002.

Table S3. Odds ratios and 95% confidence intervals of variables into a Firth logistic regression model for 3-year event-free survival.

Variable	Odds ratio	Standard error	Wald z-statistic	P-value	95% confidence interval
Transfused (vs. not transfused)	.79	.49	-.38	.70	(.23, 2.67)
Female (vs. male)	1.79	1.27	.81	13.2	(.44, 7.17)
Localized (vs. metastatic)	4.55	2.89	2.39	.02	(1.31, 15.78)
Hemoglobin at diagnosis	.97	.21	-.15	.89	(.63, 1.48)

Table S4. Odds ratios and 95% confidence intervals of variables into a Firth logistic regression model for 5-year event-free survival.

Variable	Odds ratio	Standard error	Wald z-statistic	P-value	95% confidence interval
Transfused (vs. not transfused)	.94	.51	-.11	.91	(.33, 2.69)
Female (vs. male)	1.19	.70	.30	.80	(.38, 3.75)
Localized (vs. metastatic)	4.11	2.56	2.27	.02	(1.22, 13.91)
Hemoglobin at diagnosis	.88	.16	-.68	.50	(.61, 1.27)

Table S5. Odds ratios and 95% confidence intervals of variables into a Firth logistic regression model for 3-year overall survival.

Variable	Odds ratio	Standard error	Wald z-statistic	P-value	95% confidence interval
Transfused (vs. not transfused)	.72	.49	-.49	.62	(.19, 2.70)
Female (vs. male)	2.99	2.51	1.30	.19	(.57, 15.55)
Localized (vs. metastatic)	3.30	2.24	1.76	.08	(.87, 12.46)
Hemoglobin at diagnosis	1.00	.24	.00	1.00	(.63, 1.59)

Table S6 Odds ratios and 95% confidence intervals of variables into a Firth logistic regression model for 5-year overall survival.

Variable	Odds ratio	Standard error	Wald z-statistic	P-value	95% confidence interval
Transfused (vs. not transfused)	.77	.48	-.42	.68	(.23, 2.60)
Female (vs. male)	2.22	1.63	1.09	.28	(.53, 9.33)
Localized (vs. metastatic)	2.46	1.61	1.37	.17	(.68, 8.87)
Hemoglobin at diagnosis	.90	.20	-.46	.65	(.59, 1.59)
